# Occupational dust exposure and head and neck squamous cell carcinoma risk in a population-based case–control study conducted in the greater Boston area

**DOI:** 10.1002/cam4.155

**Published:** 2013-11-04

**Authors:** Scott M Langevin, Michael D McClean, Dominique S Michaud, Melissa Eliot, Heather H Nelson, Karl T Kelsey

**Affiliations:** 1 Department of Environmental Health, University of Cincinnati College of MedicineCincinnati, Ohio; 2 Department of Environmental Health, Boston University School of Public HealthBoston, Massachusetts; 3 Department of Epidemiology, Brown UniversityProvidence, Rhode Island; 4 Masonic Cancer CenterMinneapolis, Minnesota; 5 Division of Epidemiology and Community Health, University of MinnesotaMinneapolis, Minnesota; 6 Department of Pathology and Laboratory Medicine, Brown UniversityProvidence, Rhode Island

**Keywords:** Concrete dust, epidemiology, HNSCC, leather dust, metal dust, sawdust, soot

## Abstract

Head and neck cancers account for an estimated 549,000 global cancer diagnoses each year. While tobacco use, alcohol consumption, and HPV16 infection are considered to be the major risk factors for this disease, occupational risk factors, including exposure to asbestos, have also been described, although dust exposures other than asbestos have been historically understudied. We have investigated the relationship between occupational exposures to five types of dusts, including sawdust, concrete dust, leather dust, metal dust, and chimney soot, and head and neck squamous cell carcinomas (HNSCC) in the greater Boston area. We report findings from a population-based case–control study involving 951 incident HNSCC cases and 1193 controls, frequency matched on age (±3 years), sex, and town/neighborhood of residence. Multivariable logistic regression was used to assess the association between occupational exposure to each type of dust and HNSCC, overall and by primary tumor site. After adjusting for age, sex, race, smoking, alcohol consumption, education, and HPV16 serology, laryngeal carcinoma risk increased for each decade of occupational exposure to sawdust (OR = 1.2, 95% CI: 1.0, 1.3) and metal dust (OR = 1.2, 95% CI: 1.0, 1.4); and HNSCC risk increased for each decade of occupational leather dust exposure (OR = 1.5, 95% CI: 1.2, 1.9). We have provided evidence for an association between occupational sawdust and metal dust and laryngeal squamous cell carcinoma, and leather dust and HNSCC, with increasing risk with longer duration at the exposed occupation.

## Introduction

Head and neck cancer (excluding cancers of the nasopharynx) accounts for an estimated 549,000 global cancer diagnoses each year [1], making it the 7th most common cancer worldwide (6th among men). Squamous cell carcinomas (HNSCC) makeup in excess of 90% of these cancers [2]. Smoking (and to a somewhat lesser extent, smokeless tobacco or betel quid mixtures), alcohol consumption, and HPV16 infection are widely recognized as the primary risk factors for this disease [3]. While those risk factors have garnered the majority of attention in etiologic research for HNSCC, and deservingly so, as they are attributed to the vast majority of these tumors, there is evidence that other factors may contribute to this disease, including occupational exposures [4, 5]. As manufacturing becomes progressively more globalized, the elucidation of occupational risk factors becomes increasingly critical for the formulation and implementation of adequate safety policy and procedures.

The epithelial lining of the upper aerodigestive tract is susceptible to insult from intake of environmental carcinogens via ingestion or aspiration. This is particularly relevant for airborne particles, such as dusts generated in the occupational setting. While asbestos exposure has been studied as a potential cause of HNSCC [4, 6], other occupational dusts have received little attention. Dusts, defined as small solid particles suspended in air (at least for a time) ranging from 1 to 100 *μ*m in diameter [7], are a heterogeneous group of exposures that can be either primarily organic (such as wood or leather dusts) or inorganic (such as metal dusts). Dusts may potentially exert their carcinogenic effect through induction of chronic inflammation, their intrinsic chemical properties, or by acting as carriers of other carcinogenic compounds [8]. Two forms of occupational dust, wood and leather dust, have been classified as type 1 carcinogens by the International Agency for Cancer Research (IARC), and are considered causal for cancers of the nasal cavity and paranasal sinus [8]. However, limited research has been conducted on the effects of these exposures on HNSCC, and has been particularly sparse for cancers of the oral cavity and pharynx.

As such, the goal of this study was to expand the literature on occupational exposures and head and neck cancer risk. Specifically, we have investigated the relationship between occupational exposures to five types of dusts, including sawdust, concrete dust, leather dust, metal dust, and chimney soot, and HNSCC in a population-based case–control study conducted in the greater Boston area.

## Methods

### Study population

Incident cases of head and neck squamous cell carcinoma (HNSCC; oral cavity: ICD-9 141.1−141.5, 141.8, 141.9, 143−145.2, 145.5−145.9, 149.8, 149.9; pharynx ICD-9 141.0, 141.6, 145.3, 145.4, 146, 149.0, 149.1; hypopharynx ICD-9 148; larynx: ICD-9 161) were enrolled through major teaching hospitals located in Boston, MA (Brigham and Women's Hospital, Beth Israel Deaconess Medical Center, Boston Medical Center, Dana-Farber Cancer Institute, Massachusetts Eye and Ear Infirmary, Massachusetts General Hospital, and New England Medical Center) as part of a population-based case–control study of head and neck cancer in the greater Boston area [9, 10]. For inclusion in the study, cases were required to reside in Boston or any of 162 contiguous cities and towns within ∼1 hour drive from Boston at the time of diagnosis. Control subjects with no prior history of HNSCC were selected using town records and frequency matched to cases on age (±3 years), gender, and neighborhood/town of residence. The study includes data collected from two periods of recruitment from the same population: phase I was conducted between December 1999 and December 2003 (533 cases and 685 controls) and phase II was conducted between October 2006 and June 2011 (509 cases and 567 controls); participation rates for cases and controls were 78% and 47%, respectively. Study subjects who did not provide a response for any of the five types of occupational dust exposures considered in this study (sawdust, concrete dust, leather dust, metal dust, or chimney soot) were excluded (91 cases and 59 controls), leaving 951 cases and 1193 controls for analysis. All cases and controls enrolled in the study provided written informed consent as approved by the Institutional Review Boards of the participating institutions.

### Data collection

Subjects completed a self-administered, interviewer-reviewed questionnaire that provided detailed data on sociodemographics and personal characteristics, alcohol and tobacco use, personal and family cancer history, occupational history, and other relevant dietary, health behavior, residential and medical history. In-depth occupational history was collected for each different occupation held by each study subject, including start and end dates and self-reported exposure to the dusts under consideration. Duration of work in each dust-exposed occupation was calculated for each subject by subtracting the start from end date for each occupation for which the respective occupational dust exposure was reported and summing up the total duration across all occupations.

### HPV16 serology

Serologic HPV16 testing for L1 viral protein antibody was performed on all cases and controls as a measure of past HPV16 exposure. Sandwich ELISA assays were used for detection of HPV16 antibodies as previously described [11].

### Statistical analysis

Univariable statistics for normally distributed continuous covariates (i.e., age) were assessed by two-sample *t*-test for differences between cases and controls and two-way ANOVA for differences between by primary tumor site among cases, with normality determined by the Skewness–Kurtosis test [12]. Categorical differences, by case–control status and by site, were assessed by Fisher's exact test. Kernel density plots were generated for ever-exposed subjects for total years worked at an exposed occupation for each type of occupational dust exposure (i.e., sawdust, concrete dust, leather dust, metal dust, and chimney soot) by case–control status, with differences assessed by Wilcoxon rank sum test. All tests were two sided and significance was considered where *P* ≤ 0.05.

As many occupational exposures are not fully independent, a correlation matrix was generated to evaluate the degree of correlation between occupational dust exposures and other potentially confounding exposures. Pearson's correlation coefficient (*r*) was calculated between each of the dust exposures considered in this study and occupational exposure to automobile exhaust, diesel fuel, wood smoke, and asbestos.

Unconditional multivariable logistic regression was used to estimate HNSCC risk, overall and for each respective primary tumor site (i.e., oral cavity, pharynx, larynx), associated with each type of self-reported occupational dust exposure, adjusted for age (continuous and centered at the median), sex, race (*White* vs. *non-White*), cigarette smoking (modeled both as a binary ever/never smoking term and continuously as pack-years, considered additively), alcohol consumption (categorized as: *nondrinker*, *≤14 drinks/week*, and *>14 drinks/week*), highest level of education (*high school or less* vs. *greater than high school*), and HPV16 L1 serology (*negative* vs. *positive*). For the purpose of quantifying alcohol consumption, an alcoholic beverage was defined as a 12 oz beer, 5 oz glass of wine, or 1.5 oz of liquor. Occupational dust exposure, the primary independent variable in these analyses, was separately modeled as a binary variable (*ever* vs. *never* occupationally exposed) and continuously by years of occupational exposure.

There were missing values for race (one case, one control), alcohol consumption (two cases, three controls), education (two controls), and HPV16 L1 serology (127 cases, 160 controls); data were complete for age, sex, and smoking. To compensate for the missing values in the logistic regression models, multiple imputation was employed using multivariate normal regression, based on age, sex, and smoking data; multiple imputation results in less biased findings when dealing with missing covariate data [13].

To explore the possibility of biological interaction between heightened immune surveillance and occupational dust exposure, we generated joint effects models for history of allergies and asthma with any significant occupational dust exposures from the logistic regression models, overall and by site, and then estimated the relative excess risk due to interaction (RERI), a measure of biological interaction as determined by departure from additivity [14]; separate models including multiplicative terms between each dust exposure and allergies or asthma were also generated to assess potential multiplicative interaction.

RERI estimates and corresponding 95% confidence intervals were calculated using the biological interaction tool available through EpiNET (http://www.epinet.se). All other statistical analyses were conducted in Stata 11 (College Station, TX).

## Results

Relative to control subjects, cases were slightly younger by an average of 1.3 years (*P* = 0.01), and differed with respect to smoking (*P* < 0.001), alcohol consumption (*P* < 0.001), level of education (*P* < 0.001), and HPV16 exposure (as determined by HPV16 L1 serology; *P* < 0.001). Among the cases, there were significant differences across primary tumor sites (oral cavity, pharynx, and larynx) for age (*P* = 0.04), sex (*P* < 0.001), smoking habit (*P* < 0.001), alcohol consumption (*P* = 0.01), education level (*P* = 0.001), and HPV16 serology (*P* < 0.001). A detailed description of the study population is provided in Table 1.

**Table 1:** Description of the study population by case–control status and primary tumor site.

	Controls (*n*=1193)	HNSCC (*n*=951)	*P*_difference_	HNSCC by site			*P*_difference_
	
				Oral cavity (*n*=355)	Pharynx (*n*=437)	Larynx (*n*=159)	
Age, mean years (*σ*)	60.9 (11.0)	59.6 (11.3)	0.01^1^	59.9 (12.7)	58.7 (10.4)	61.3 (10.3)	0.04^2^
Sex, *n* (%)							
Male	868 (72.8)	703 (73.9)	0.56^3^	228 (64.2)	255 (81.2)	120 (75.5)	<0.001^4^
Female	325 (27.2)	248 (26.1)		127 (35.8)	82 (18.8)	39 (24.5)	
Race, *n* (%)							
White	1074 (90.1)	869 (91.5)	0.30^3^	323 (91.0)	403 (92.4)	143 (89.9)	0.56^4^
Non-White	118 (9.9)	81 (8.5)		32 (9.0)	33 (7.6)	16 (10.1)	
Cigarette smoking							
Never smoker, *n* (%)	480 (40.2)	235 (24.7)	<0.001^3^	97 (27.3)	118 (27.0)	20 (12.6)	<0.001^4^
Ever smoker, *n* (%)	713 (59.8)	716 (75.3)		258 (72.7)	319 (73.0)	139 (87.4)	
Pack-years, median (range)	25 (0.1–200)	34 (0.1–203)	<0.001^5^	35 (0.1–147)	31 (0.4–203)	41 (0.2–150)	0.001^6^
Alcohol consumption, *n* (%)							
Nondrinker	149 (12.5)	88 (9.3)	<0.001^3^	36 (10.2)	34 (7.8)	18 (11.3)	0.01^4^
≤2 drinks per day	753 (63.3)	430 (45.3)		166 (47.0)	212 (48.5)	52 (32.7)	
>2 drinks per day	288 (24.2)	431 (45.4)		151 (42.8)	191 (43.7)	89 (56.0)	
Highest level of education, *n* (%)							
High school or less	327 (27.5)	389 (40.9)	<0.001^3^	158 (44.5)	151 (34.6)	80 (50.3)	0.001^4^
Greater than high school	864 (72.5)	562 (59.1)		197 (55.5)	286 (65.5)	79 (49.7)	
HPV16L1 serostatus, *n* (%)							
Negative	965 (93.4)	584 (70.5)	<0.001^3^	264 (86.8)	206 (52.8)	114 (84.4)	<0.001^4^
Positive	68 (6.6)	245 (29.6)		40 (13.2)	184 (47.2)	21 (15.6)	

1 Two-sample *t*-test for difference between cases (all HNSCC) and controls.

2 ANOVA for difference across primary tumor sites (cases only).

3 Fisher's exact test for difference between cases (all HNSCC) and controls.

4 Fisher's exact test for difference across primary tumor sites (cases only).

5 Rank-sum test for difference between cases (all HNSCC) and controls.

6 Kruskal–Wallis test for difference across primary tumor sites (cases only).

**Figure 1:**
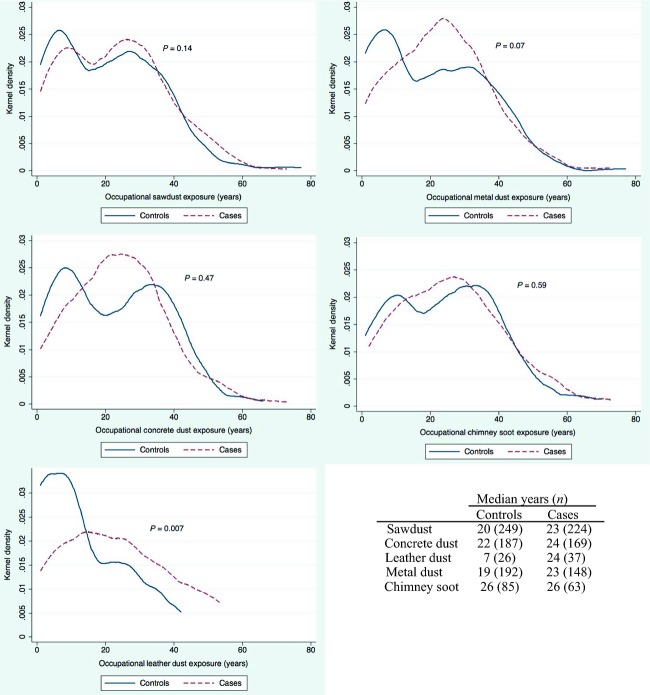
Distribution of duration of occupational dust exposure in years by case–control status among study subjects reporting ever having been exposed. The plots represent the kernel density of each type of dust exposure for cases and controls, and median years of occupational exposure are presented in the table in the lower right corner. The *P*-value for difference between cases and controls was determined by Wilcoxon rank sum test and is presented on each respective corresponding plot.

Among subjects reporting an occupational exposure to each respective type of dust, the duration of occupation with leather dust exposure was significantly longer among cases than controls (*P* = 0.007), with a median exposure of 24 years among 37 cases versus 7 years among 26 controls (Fig. 1). Exposed cases also experienced a marginally longer duration of occupational exposure to metal dust relative to controls (*P* = 0.07).

As a number of different exposures may be common to an occupation, we constructed a correlation matrix to explore the relationship between dust exposures and other select occupational exposures (particularly those containing polycyclic aromatic hydrocarbons) that could potentially influence HNSCC risk (Table 2). With the exception of leather dust, moderate positive correlation (ranging from 0.30 to 0.65) was observed between occupational dusts, diesel fumes, automobile exhaust, wood smoke, woodworking, and asbestos.

**Table 2:** Pearson correlation matrix of self-reported occupational exposure to dusts, automotive exhaust, diesel fumes, wood smoke, woodworking, and asbestos.

	Sawdust	Concrete dust	Leather dust	Metal dust	Chimney soot	Auto exhaust	Diesel fuel	Wood smoke	Woodworking	Asbestos
Sawdust	1.00									
Concrete dust	0.57	1.00								
Leather dust	0.13	0.15	1.00							
Metal dust	0.39	0.39	0.22	1.00						
Chimney soot	0.34	0.37	0.22	0.30	1.00					
Automotive exhaust	0.38	0.41	0.15	0.32	0.32	1.00				
Diesel fuel	0.37	0.45	0.16	0.33	0.34	0.60	1.00			
Wood smoke	0.45	0.44	0.20	0.32	0.48	0.36	0.37	1.00		
Woodworking	0.65	0.50	0.15	0.37	0.30	0.35	0.31	0.49	1.00	
Asbestos	0.46	0.49	0.14	0.37	0.38	0.35	0.35	0.33	0.38	1.00

In the multivariable models (Table 3), there was a borderline association between a history of occupational sawdust exposure and laryngeal squamous cell carcinoma (OR = 1.4, 95% CI: 1.0, 2.2) with an increase in risk for each decade of exposure (OR = 1.2, 95% CI: 1.0, 1.3), after adjusting for age, sex, race, smoking, alcohol consumption, education, and HPV16 serology. For each decade of occupational exposure to leather dust, there was increased risk of HNSCC (OR = 1.5, 95% CI: 1.2, 1.9) and pharyngeal squamous cell carcinoma (OR = 1.7, 95% CI: 1.2, 2.2), after adjustment for age, sex, race, smoking, alcohol consumption, education, and HPV16 serology; point estimates were also elevated (although nonsignificant) for cancers of the oral cavity and larynx for each successive decade of exposure. There was also a borderline increased risk for laryngeal cancer associated with each decade of metal dust exposure (OR = 1.2, 95% CI: 1.0, 1.4).

**Table 3:** Head and neck squamous cell carcinoma risk from any lifetime exposure to occupational dusts and by duration at exposed occupation, overall and by primary tumor site.

Occupational dust	HNSCC		Oral cavity		Pharyngeal		Laryngeal	
			
	*n*_cases_/*n*_control_	OR^1^ (95% CI)	*n*_cases_/*n*_control_	OR^1^ (95% CI)	*n*_cases_/*n*_control_	OR^1^ (95% CI)	*n*_cases_/*n*_control_	OR^1^ (95% CI)
Sawdust								
Never exposed	708/927	Reference	277/927	Reference	327/927	Reference	104/927	Reference
Ever exposed	243/266	1.0 (0.8, 1.2)	78/266	0.9 (0.6, 1.2)	110/266	0.9 (0.7, 1.2)	55/266	1.4 (1.0, 2.2)
Per decade of exposure	224/249	1.0 (0.9, 1.1)	71/249	0.9 (0.8, 1.0)	99/249	1.1 (1.0, 1.2)	54/249	1.2 (1.0, 1.3)*^2^
Concrete dust								
Never exposed	768/991	Reference	294/991	Reference	345/991	Reference	129/991	Reference
Ever exposed	183/202	0.9 (0.7, 1.2)	61/202	0.9 (0.6, 1.3)	92/202	1.1 (0.8, 1.5)	30/202	0.9 (0.5, 1.4)
Per decade of exposure	169/187	1.0 (0.9, 1.1)	58/187	0.9 (0.8, 1.1)	84/187	1.0 (0.9, 1.2)	27/187	1.0 (0.9, 1.2)
Leather dust								
Never exposed	911/1160	Reference	343/1160	Reference	417/1160	Reference	151/1160	Reference
Ever exposed	40/33	1.2 (0.7, 2.1)	12/33	0.9 (0.5, 1.9)	20/33	1.3 (0.7, 2.6)	8/33	1.2 (0.5, 2.9)
Per decade of exposure	37/26	1.5 (1.2, 1.9)*	11/26	1.3 (0.9, 1.8)	19/26	1.7 (1.2, 2.2)*	7/26	1.5 (1.0, 2.2)
Metal dust								
Never exposed	790/988	Reference	303/988	Reference	361/988	Reference	126/988	Reference
Ever exposed	161/205	0.9 (0.7, 1.1)	52/205	0.8 (0.5, 1.1)	76/205	0.9 (0.6, 1.3)	33/205	1.1 (0.7, 1.7)
Per decade of exposure	148/192	1.0 (0.9, 1.1)	47/192	0.9 (0.8, 1.1)	70/192	1.0 (0.9, 1.2)	31/192	1.2 (1.0, 1.4)
Chimney soot								
Never exposed	880/1103	Reference	335/1103	Reference	400/1103	Reference	145/1103	Reference
Ever exposed	71/90	0.9 (0.6, 1.3)	20/90	0.7 (0.4, 1.2)	37/90	1.2 (0.7, 1.9)	14/90	1.0 (0.5, 1.9)
Per decade of exposure	63/85	1.0 (0.9, 1.1)	18/85	0.9 (0.7, 1.1)	32/85	1.1 (0.9, 1.3)	13/85	1.0 (0.8, 1.2)

* *P*≤0.05.

1 Adjusted for age, gender, race, smoking, alcohol consumption, education, and HPV16 exposure.

2 Actual estimated value of the lower confidence interval is 1.0004 (*P*=0.049).

In response to the moderate correlations observed between sawdust exposure and automotive exhaust (*r* = 0.38), diesel fuel (*r* = 0.37), wood smoke (*r* = 0.45), woodworking (*r* = 0.65), and asbestos (*r* = 0.46), we additionally ran the models for sawdust and metal dust and laryngeal cancer including each of the respective aforementioned exposures as a covariate. None of these significantly altered the magnitude or significance of the laryngeal cancer risk estimates for sawdust or metal dust (data not shown) and therefore were not included in the final model for reasons of parsimony.

To test the robustness of our findings, we performed sensitivity analyses for each significant exposure and site in which we reanalyzed the data alternatively coding smoking categorically based on pack-year tertiles in the controls (*never smoker*, *0.1*–*16.0 pack-years*, *16.1*–*36.0 pack-years*, and *>36.0 pack-years*) and a continuous treatment of average drinks per week, in varying combinations; point estimates and significance levels remained similar to those observed in the original models (data not shown). We additionally reanalyzed the model for leather dust exposure and pharyngeal cancer, restricted to only oropharyngeal cancers (excluding 61 hypopharyngeal cases); point estimates were similar to those for all pharyngeal cases (OR = 1.8, 95% CI: 1.3, 2.5).

No effect modification was observed (on the additive or multiplicative scale) between history of asthma, allergies, or major HNSCC risk factors (heavy smoking/drinking or HPV16 serostatus) and occupational exposure to any of the dusts under consideration (data not shown).

## Discussion

While IARC considers both wood dust and leather dust to be causal for sinonasal cancers [8], the literature remains sparse for other cancers of the upper aerodigestive tract, especially for oral and pharyngeal cancers. Here, we provide evidence from a large, population-based case–control study of HNSCC for occupational sawdust exposure as a risk factor for laryngeal squamous cell carcinoma and occupational exposure to leather dust as a risk factor for HNSCC, each exhibiting a dose–response. We additionally observed a borderline dose–response relationship between occupational metal dust exposure and laryngeal carcinoma. No significant associations were observed between occupational exposure to concrete dust or chimney soot and HNSCC, overall or for any of the specific sites (i.e., oral cavity, pharynx, or larynx). To our knowledge, this is the first report of a significant association between leather dust and HNSCC.

Occupational wood dust exposure has long been associated with adverse health outcomes, with the highest exposure levels to wood dust occurring in the construction and furniture industries [8]. The nature of the association between wood dust exposure and HNSCC has been somewhat controversial in the literature, with a number of reports examining this relationship, particularly for laryngeal and hypopharyngeal cancers (much less so for cancers of the oral cavity and oropharynx), with mixed results. Our observed association between sawdust and laryngeal cancer is corroborated by some of the reports on this relationship [15, 16] or with wood-related occupations [17–19]. However, not all studies are in agreement, with a number finding no association [20–28], and with one even reporting a significant inverse association [29]. Getting at the true risk of laryngeal cancer (if any) that is associated with wood dust is complicated by the heterogeneity of the exposure across studies. The source and content of wood dust vary by region [8] and no reliable data presently exist regarding health risks from dust generated from different species of wood. This indicates a need for better, more detailed exposure assessments in occupational studies of wood dust, in order to bolster our ability to identify workers with the highest levels of risk.

Over the past half-century, there has been a shift in leather industries from developed to developing countries [8]. Although difficult to precisely quantify due to subcontracted home-based labor practices, it is estimated that several million people globally work in the leather industry [8], underscoring the need for increased research into the possible health effects of related exposures. Leather dust can vary greatly in properties, dependent on the chemicals used in the tanning or processing or the material, and like wood dust can be regionally variable. Chromium(III) is often used in the tanning process, and can range from 0.1% to 4.5% of the total leather dust weight, some with detectable levels of chromium(VI) [30], which is considered by IARC to be carcinogenic to humans (group 1) [31].

We observed associations between leather dust and HNSCC, overall and for pharyngeal cancer. Although not significant, point estimates were similarly elevated for cancers of the oral cavity and larynx. While this is the first study, to our knowledge, to report this association, a small handful of others has also investigated this relationship [23, 24, 29]. These studies, however, all suffered greatly from a lack of statistical power, with very few exposed subjects in any of them. Regardless of the low numbers, Gustavsson et al. reported consistent elevated relative risks for HNSCC, overall and by site (OR point estimates ranging from 2.06 to 2.83). Additionally, a multicountry European study of occupation and cancers of the hypopharynx and larynx found increased risk of laryngeal cancer among shoe finishers [17], although not for those occupationally involved with shoe making or repair.

Several toxic metals or metalloids (inorganic compounds) are considered by the International Agency for Cancer Research (IARC) to be definite or probable carcinogens, including nickel, cobalt, lead, vanadium, beryllium, arsenic, and chromium [32–34]. Metals are characterized by their biopersistence [35], and represent a broad class of elements and alloys. While the carcinogenic mechanisms for most metals and metalloids are not as well understood as they are for organic carcinogens, it is believed that metals may exert their carcinogenicity through means including (but not limited to) epigenetic alterations, deregulation of cellular proliferation and metabolism, aberrant activation of signal transduction pathways, generation of reactive oxygen species, and induction of hypoxia pathways [36], or by competitive binding with enzyme-associated metals [37].

Although our observed relationship between metal dust exposure and laryngeal cancer was marginal, the finding is strengthened by the apparent dose–response nature of the association. These findings are further corroborated by several independent reports in the literature that have also identified occupational exposure to metal dust as a risk factor for laryngeal carcinoma [27, 29, 38], with others finding increased risk among those in metal working occupations [17, 18, 39]. Although the literature is relatively sparse, our findings, paired with others on this topic, indicate a need for additional research into the impact of these exposures on head and neck cancer risk.

The major strengths of this study include the use of well-annotated population-based data with a relatively large number of cases and collection of detailed occupational history, allowing us to generate well-controlled, generalizable risk estimates. Weaknesses may include the self-reported nature of the exposures, which could lead to differential misclassification or possibly a recall bias. It seems, however, improbable that if we were to see a recall bias, it would only be observed for some forms of dust but not others, as sawdust, leather dust, and metal dust are not likely to be widely recognized as carcinogens by the general public. Additionally, despite our relatively large sample size (by head and neck cancer standards), we are still underpowered to detect associations with some of the more rare exposures, particularly when looking at specific sites (i.e., oral cavity, pharynx, or larynx) or by duration. Also, the nature of our data does not provide us with frequency or intensity of each exposure, although we have data indicating duration of employment at each exposed occupation that we were able to use as a surrogate for dose. We also were able to control for self-reported asbestos exposure, but cannot rule out some residual confounding.

To summarize, we have provided evidence for an association between occupational sawdust and metal dust exposures and laryngeal squamous cell carcinoma, and leather dust exposure and HNSCC, which appeared particularly strong for pharyngeal carcinoma. While a relatively small proportion of head and neck cancers are likely attributable to occupational exposures, mitigation of such risks can still result in a sizable reduction in absolute number of cases at the global level. Continued efforts, such as those detailed in this report, at identifying occupational risk factors for cancers of the upper aerodigestive tract will have clear implications on risk assessment, formulation of workplace policy, and appropriate engineering of safeguards to help reduce the incidence of occupational cancers.
